# Total Knee Arthroplasty In Patients With Parkinson's Disease- A Critical Analysis of Available Evidence

**DOI:** 10.2174/1874325001711011087

**Published:** 2017-09-30

**Authors:** Munis Ashraf, Sruthi Priyavadhana, Senthil Nathan Sambandam, Varatharaj Mounasamy, Om Prakash Sharma

**Affiliations:** 1 Department of Orthopaedics, K.G. Hospital and Postgraduate Medical Institute, Arts College Road, Coimbatore 641018, Tamil Nadu, India; 2 Medical student Saveetha medical college Chennai, Tamil Nadu, India; 3 VCU Medical Center Ambulatory Care Center, 417 North 11th Street, Richmond, Virginia, USA; 4 Orthopedic surgeon Essentia Health St.Mary’s Detroit Lakes Clinic, Minnesota, USA

**Keywords:** Total knee arthroplasty, Parkinson’s Disease, Hoehn yahr, Flexion contracture, Physical therapy, Columbia classification

## Abstract

**Background:**

In this era of modern medicine, there is an increase in life expectancy and thereby an ageing population. Among this group one of the most common neurological disorder is Parkinson disease and one of the most common operation done in elderly population is a total joint arthroplasty. But total joint arthroplasty in Parkinson disease is a relatively uncommon entity. There is sparse literature available with regards to total knee arthroplasty (TKA) in Parkinson disease. This review focusses on the necessity, complications and previous experiences on TKA in PD based on the literature available.

**Method:**

The review was conducted after a series of advanced search in the following medical databases; Pub med, Biomed central, Cochrane and Google scholar for articles related to total knee replacement in patients with Parkinson’s disease. The following keywords were used; Total knee arthroplasty, Parkinson’s disease, Hoehn and Yahr, Flexion Contracture.

**Results:**

The review indicates that the functional outcome is comparable to that of controls in immediate post-operative phase, one year and three-year phase, but the long term functional outcome seems to deteriorate significantly.

**Conclusion:**

Total knee arthroplasty can serve as an effective tool in alleviating pain in short term as well as long term periods, whereas the functional outcome seems to deteriorate post operatively on a long-term basis. Nevertheless, TKA in PD is a challenging situation, thereby necessitating a holistic approach with the efforts from various specialists needed at each stage to ensure a successful operation.

## 1 INTRODUCTION

Parkinson’s disease (PD) is a neurodegenerative disorder affecting approximately 4 million people worldwide. The disease is due to the decrease in the dopaminergic activity in the nigro striatal pathway leading to a dysfunction of the basal ganglia and is characterised by motor disturbances and cognitive impairments [1]. The mean age of onset is 55 years [2]. With the improvement in life expectancy, the prevalence of Parkinson’s disease is projected to be on the rise and thereby a significant increase in number of total knee arthroplasty (TKA) to be performed in PD. Therefore, it is of paramount importance for the orthopaedic surgeon to have clarity and knowledge in the approach of TKA in PD amidst varying results as recorded in available literature till date. This review tries to delineate the common problems encountered, factors influencing the outcome and considerations for TKA in PD based on the available literature.

## 2 FACTORS AFFECTING THE OUTCOME FOR TOTAL KNEE ARTHROPLASTY IN PARKINSON’S DISEASE

### 2.1 General Considerations

#### 2.1.1 Age: 

A review of epidemiology in knee arthroplasty highlights that there has been a fivefold increase in the rates of primary TKA since 1980, primarily due to an ageing population [3]. The mean age at the time of presentation varies from 70-73 years in the available literature [4-7]. Higher age group is associated with increased mortality and ironically a decrease in revision rates [8].

#### 2.1.2 Gender:

The utilisation rates of arthroplasty seems to be equal in males and females. In PD post TKA, males are seen to have an increase in mortality whereas females are associated with an increase duration of hospital stay [8].

#### 2.1.3 Clinical features:

Selection of patients with a preoperative flexion contracture, dementia and medical co morbidities like cardiovascular disease are associated with increased mortality and decreased functional outcome post TKA [8].

#### 2.1.4 Stage of Parkinson’s disease:

A pre-operative staging of disease is essential to determine to what extent the PD symptoms can worsen postoperatively in a given time. A scoring system would help in delineating patients who can be candidates for TKA. PD patients with impaired righting reflexes and physical instability are a contraindication for TKA. Patients with a Modified Hoehn and Yahr stage of disease less than 3 can be operated upon after taking into account the associated comorbidities. Modified Hoehn and Yahr staging (Table **1**), Columbia classification (Table **1a**) were used in the studies [5, 6, 9, 10].

#### 2.1.5 Bone Mineral Density:

Patients with PD have a positive correlation with reduced bone density and are prone to vitamin D deficiency. The bradykinesia and rigidity results in the lack of spontaneous activity and muscle disuse. This in turn predisposes for an increased osteoclast mediated calcium resorption. The reduced bone mass can lead to an increased risk of peri prosthetic fracture in patients with PD. Treatment with bisphosphonates and teriparatide has been tried, however there are no consensus in this regard. However, it is imperative that bone density is assessed in patients with PD and treated appropriately pre-operatively [11].

The authors feel that the indication for TKA in PD is almost similar to that of TKA in general population, mostly due to the degenerative osteoarthritis. In addition, x ray studies have shown that the medial compartment is more commonly affected than the lateral compartment [6].

### 2.2 Intra Operative Considerations

#### 2.2.1 Anaesthesia:

From the available studies the use of general anaesthesia is to be avoided as the chances of post-operative confusion and psychological co morbidities is higher. More specifically, the use of levodopa and bromocriptine is known to cause orthostatic hypotension and therefore it is suggested that these medications are administered at low doses. Propofol is widely used for induction in general anaesthesia for neurodegenerative conditions but it is associated with spontaneous involuntary movements in patients with Parkinson’s. There occurs an increased sensitivity of the myocardium to the catecholamines when halothane is used in patients receiving levodopa. Due to the risk of exacerbation of extra pyramidal symptoms, anti-emetics like phenothiazine and metoclopramide are best avoided. Factoring in on these inherent complications of general anaesthesia in patients with PD, the use of regional anaesthesia spinal, epidural / combined has been suggested to be a safer option [11].

#### 2.2.2 Implants:

Implant selection is of great importance as it can decide the functional outcome of TKA in PD. The most important consideration while selecting the implant is the role of extensor mechanism of PD patients. The extensor mechanism is prone to disruption due to rigid hamstrings or severe flexion contracture and this can lead to implant dislocation. Condylar constrained, hinged knee device or cruciate retaining device is used to overcome this [6, 12]. No single design of TKA is suitable for every patient, the design should be chosen with the conditions of the patient and the comfort of the operating surgeon in mind.

#### 2.2.3 Unilateral versus Bilateral:

The scope of a bilateral TKA in PD is less. Duffy and Trousdale have found that a bilateral TKA is associated with a poor knee society functional score (KSFS) at 3 years and further decline in KSFS score at end of five years. With unilateral TKA there is an improvement in KSFS score after three years but a decline in KSFS score is noted at the end of five years [6].

#### 2.2.4 Blood loss:

Till date there is no evidence in literature stating the blood loss and its role in TKA for PD patients.

### 2.3 Post-Operative Considerations

#### 2.3.1 Pain management:

Unlike pain management of TKA in general population, the use of opioids is avoided in PD. The reason for avoidance of opioids is its ability to significantly depress the CNS in an already co-existing cognitive dysfunction. Patients with neurogenerative diseases are likely to be on monoamine oxidase inhibitors (MAOI) and the use of opioids in conjunction with the MAOI’s can lead to the development of serotonin syndrome. Hence, the opioids are contraindicated [11] and the anti-inflammatory medications are used for pain management post operatively in patients with normal renal functions [13].

#### 2.3.2 Infections:

The occurrence of superficial skin infection as well as deep infection are noted in studies [6, 8]. This points to a strict vigil that needs to be maintained during wound checks and sufficient antibiotic prophylaxis to be administered.

#### 2.3.3 Physical therapy:

The initial studies on TKA in PD have proven that a delay in initiation in physical therapy results in flexion contracture, quadriceps rupture and a poor functional outcome. There should be a gradual increase in initiation of physical therapy as conventional post-operative TKA physical therapy protocols can result in disruption of extensor mechanism (14,15).

#### 2.3.4 Musculoskeletal issues:

As noted in many of the earlier studies, the occurrence of flexion contracture, extensor mechanism disruption due to a rigid hamstring are a few musculoskeletal complications which can occur post operatively [4, 5, 9]. Proper rehabilitation methods are needed to be in place to check these complications [15].

#### 2.3.5 Worsening of Parkinson’s Disease:

Most of the studies indicate an increase in severity of the disease post operatively on a long-term basis [7, 8]. This change can be detected by worsening cognition, impaired righting reflexes or an increase in muscle imbalance [16].

#### 2.3.6 Thromboprophylaxis:

Vince *et al* [5] had reported incidence of DVT and pulmonary embolism, hence the initiation of mechanical thromboprophylaxis with incentive spirometry, chest physiotherapy, pneumatic compression devices is essential. However, it is to be noted that there are no studies specifically indicating the rates of thromboembolic episodes in patients with PD. The authors suggest that for patients with PD, DVT prophylaxis measures should be practiced just as in TKA in general population.

## 3 THE FUNCTIONAL OUTCOME OF TKA IN PD

The functional outcome was quantified in three studies. Among the three studies, the study conducted in Dundee, UK was the sole exclusive outcome analysis study [7]. This study had used the knee society score and knee society function score. The other two studies used the knee society score [5, 6] (Table **2**).

These reports indicate that the functional outcome is comparable to that of controls in immediate post-operative phase, one year and three-year phase, but the long term functional outcome seems to deteriorate significantly. The deterioration in functional outcome can be attributed to the progression of the Parkinson’s disease and not the operation per se.

## 4 COMPLICATIONS RELATED TO TKA IN PD

Some of the complications post TKA in PD based on available literature is discussed below and given in Table **3**.

### 4.1 Flexion Contracture

The most highlighted complication in literatures is of the development of a flexion contracture in post TKA patients. This can be viewed in two aspects; first one being a delayed initiation of physical therapy and second aspect being development of flexion contracture in PD patients who had pre-operative muscle imbalance and ataxia [4, 5]. Flexion contracture post operatively was observed only in three studies (Oni and Mackenney in 1985, Insall *et al* 1989 and Shah *et al* 2004). In all these three studies initiation of physical therapy was delayed by atleast a week post operatively; whereas in other studies there was prompt early mobilisation which resulted in no flexion contracture post operatively, thereby highlighting the need for early mobilisation postoperatively. A study by Shah *et al* [9] in 2004 demonstrated the potential role of injecting Botulinum toxin type A into the muscle to relieve flexion contracture. Presence of flexion contracture postoperatively can significantly affect the functional capacity and range of movements in patients sometimes necessitating revision in such patients [4, 5].

## 5 INFECTIONS

Superficial necrosis and wound infection was seen in five patients in the study conducted by G.P.Duffy and Robert Trousdale [6] that led to a delay in the outcome process. The infection rates in PD post TKA is comparable with that of the controls as stated in the nationwide registry of Finland study [8].

## CONCLUSION

Total knee Arthroplasty is not an absolute contraindication but can be a relative indication in patients with Parkinson’s disease where conservative line of management has failed. Preoperative assessment, collaborated efforts of specialists from various disciplines, appropriate surgical prosthesis design, early physiotherapy aids to prevent postoperative complications and a reduced hospital stay. TKA can serve as an effective tool in alleviating pain in short term as well as long term, whereas the use of TKA for the functional outcome rather seems to decrease post operatively on a long-term basis. Further studies focussing on functional outcome in post TKA PD patients with parameters like progression of disease, severity of disease and associated medical comorbidities should be compared and studied with matched controls over a long term for better understanding of tolerance of TKA in PD. TKA in PD is a challenging situation, thereby necessitating a holistic approach with the efforts from various specialists needed at each stage to ensure a successful operation.

## Figures and Tables

**Table 1:** Staging of parkinsons disease.

**Modified Hoehn And Yahr Staging**	
**STAGE**	DESCRIPTION
**0**	No sign of disease
**1**	Unilateral disease
**1.5**	Unilateral plus axial involvement
**2**	Bilateral disease without involvement of balance
**2.5**	Mild bilateral disease, with recovery on pull test
**3**	Mild to moderate bilateral disease, some postural instability, physically independent
**4**	Severe disability, still able to walk or stand unassisted
**5**	Wheel chair bound or bed ridden unless aided

**Table 1a:** Staging of parkinsons disease.

**Columbia Classification**	
**STAGE**	DESCRIPTION
**1**	Unilateral involvement with no functional impairment.
**2**	Bilateral or midline involvement without impairment of balance
**3**	Impaired righting reflexes
**4**	Severely disabling disease
**5**	Confined to bed or wheelchair

**Table 2:** Review of literature.

Study	Published Year	Total Knees	Study Type	Scoring System	Functional Outcome Scoring	Complications
Oni &Mackeney [4]	1985	3	Case Series	Not used	Not used	Flexion contracture Quadriceps tendon rupture Confusion
Insall *et al* [5]	1989	13	Retrospective analysis	Hoehn and Yahr Classification	Hospital for special surgery knee score	Flexion contracture. Bilateral patellar fracture.confusion. patellar subluxation. Deep vein thrombosis. Pulmonary embolism. Urinary tract infection, skin necrosis. intestinal ileus.
Fast *et al* [10]	1994	1	Case report	Hoehn and Yahr Classification	Not used	None
Duffy and Trousdale [6]	1996	33	Retrospective analysis	Columbia Classification	Knee Society Score (KSS)	Confusion, patellar subluxation, deep vein thrombosis, superficial infection, myositis ossificans, wound necrosis, respiratory tract infection,reoperation deep infections, patella fracture
*Erceg, Maricevic* [11]	2000	1	Case report	Not used	Not used	Recurrent posterior tibial dislocation
Shah *et al* [9]	2004	1	Case report	Columbia Classification	Not used	Flexion contracture, diabetic coma, pneumonia, urinary tract infection
Tinning *et al* [7]	2013	32	Case control study	Not used	Knee Society Score (KSS) and Knee Society Function Score (KSFS)	Functional outcome study
Jämsen *et al* [8]	2014	567	National Joint registry data	Not used	Not used	Surgical site infection deep infection increased mortality prolonged hospital stay

**Table 3:** Complications.

**Local**	**Systemic**
**Flexion contracture** [4, 5]	**Neurological: confusion** [6]
**Extensor mechanism disturbances** [4, 5] **(quadriceps tendon rupture)**	**Gastrointestinal: postoperative ileus** [5]
**Patellar fracture** [5], **patella subluxation** [6]	**Pulmonary: respiratory tract infections** [6], **pulmonary embolism** [5].
**Wound necrosis** [4, 6]	**Renal: uremia** [4]
**Surgical site infection and deep infection** [6]	**Vascular: deep vein thrombosis** [6]
**Recurrrent posterior tibial dislocation** [17]	**Genitourinary: urinary retention** [6] **and urinary tract infections.**
	**Increased mortality and hospital stay** [8]

## LIST OF ABBREVIATIONS

PD: = Parkinsons disease
TKA: = Total knee arthroplasty
DVT: = Deep Vein Thrombosis
KSFS: = Knee society functional score
MAOI: = Mono amine oxidase inhibitors
